# Independent effects of posttraumatic stress disorder diagnosis and metabolic syndrome status on prefrontal cortical thickness and subcortical gray matter volumes

**DOI:** 10.1080/19585969.2023.2237525

**Published:** 2023-07-27

**Authors:** Hilmar Klaus Luckhoff, Stefan du Plessis, Heuvel Leigh van den, Robin Emsley, Soraya Seedat

**Affiliations:** a Department of Psychiatry, Faculty of Medicine and Health Sciences, Stellenbosch University, Cape Town, South Africa; b SAMRC Genomics and Brain Disorders Unit, Department of Psychiatry. Faculty of Medicine and Health Sciences, Stellenbosch University, Cape Town, South Africa

**Keywords:** Metabolic syndrome, post-traumatic stress disorder, prefrontal cortex, triglycerides, ventral diencephalon

## Abstract

**Introduction:**

Posttraumatic stress disorder (PTSD) and metabolic syndrome (MetS) are associated with overlapping brain structural differences. These often involve brain structures involved in the regulation of appetite, food intake, satiety, and reward processing. We examined the individual and interactive effects of PTSD diagnosis and MetS on cortical thickness and subcortical gray matter volumes in patients with PTSD (*n* = 104) compared to trauma-exposed controls (*n* = 97).

**Methods:**

Multivariate models were constructed for FreeSurfer-generated prefrontal cortical thickness and subcortical gray matter regions-of-interest (ROIs) to explore the effects of PTSD diagnosis and MetS as predictors, adjusting for relevant socio-demographic and clinical covariates. Individual prefrontal cortical and subcortical limbic ROIs were also selected based on *a priori* evidence of their involvement in both PTSD and MetS.

**Results:**

The mean age of the sample (*n* = 201; 78% female) was 41.6 (SD, 13.1) years. PTSD and MetS status showed independent associations with prefrontal cortical thickness and subcortical gray matter volumes across multiple ROIs, adjusting for age, sex, scanner sequence, alcohol, and tobacco use.

**Conclusions:**

PTSD and MetS are independently associated with brain structural differences, including thinner prefrontal cortical thickness and smaller subcortical gray matter volumes, across multiple ROIs implicated in the hedonic and homeostatic regulation of food intake.

## Introduction

Metabolic syndrome (MetS) and post-traumatic stress disorder (PTSD) are often comorbid and share overlapping neurobiological and clinical features (Aaseth et al. 2019). Meta-analyses have reported a higher prevalence of MetS in patients with PTSD compared to the general population (Bartoli et al. 2013; Rosenbaum et al. 2015). PTSD is also a known risk factor for the development of MetS (Farr et al. 2015; Francis et al. 2015).

This overlap can be explained in part by the involvement of shared pathogenic mechanisms common to both conditions. These include genetic factors (Misganaw et al. 2022; Pathak et al. 2022), chronic inflammation (Vitaliano et al. 2002; Levine et al. 2014), oxidative stress (Bonomini et al. 2015; Treviño et al. 2015), hypothalamic-pituitary-adrenal (HPA) axis dysfunction (Yehuda et al. 1995; Kibler et al. 2014), and impaired immune regulation (Hoge et al. 2009; Lindqvist et al. 2014; Brudey et al. 2015). Similar mechanisms are therefore implicated in the clinical deterioration of PTSD and the development of adverse cardiovascular events associated with MetS (Michopoulos et al. 2016). Nevertheless, extrinsic factors, such as environmental exposures, are thought to modulate the effects of biological factors on the expression of PTSD and MetS, thereby contributing to a heterogeneous clinical presentation (Rosenbaum et al. 2015; Womersley et al. 2022).

PTSD is associated with structural brain changes, including cortical thinning and subcortical volume loss, at least some of which overlap with those described for MetS (Song et al. 2015; Wolf et al. 2016). For example, obesity is a risk factor for fronto-temporal cortical thinning and subcortical gray matter volume loss in the general population (Salat et al. 2004; Hassenstab et al. 2012; Marqués-Iturria et al. 2013; Veit et al. 2014; Walhovd et al. 2014; Kaur et al. 2015). Smaller gray matter volumes (Lu et al. 2021) for several cortical regions have been described, including the orbitofrontal and temporal cortices (Kotkowski et al. 2019). Subcortical volume loss has also been reported in adolescents with MetS (Yau et al. 2012). In a similar vein, thinner cortices have been described for patients with other individual MetS risk factors, including hypertension (Leritz et al. 2011; Alosco et al. 2014), insulin resistance (Brundel et al. 2010; Leritz et al. 2011; Tchistiakova et al. 2014; Chen et al. 2015), and dyslipidaemia (Leritz et al. 2011).

However, several aspects concerning the relationships between PTSD, MetS, and brain structure have yet to be clarified. First, to what extent the associations between MetS and brain structure are specific to PTSD or rather mirror those described in the general population remains unclear. Second, whether structural brain differences described for PTSD and MetS are generalised versus region-specific remains incompletely understood. Third, the associations of PTSD severity with and differential effects of individual MetS features on brain structure remain incompletely elucidated.

Very few studies have examined whether the associations of PTSD diagnosis and MetS with brain structure are independent, additive, or synergistic, or examined the moderating effects on the expression of these brain structural changes. For example, Wolf et al. (2016) conducted a whole-brain analysis in a sample of war veterans, and found that PTSD diagnosis mediated the effects of MetS as a predictor of thinner cortical thickness. More research is however needed to better understand these relationships.

In response to this knowledge gap, the present study examined the associations of MetS with brain structure in patients with PTSD compared to trauma exposed controls (TEC). For our primary objective, we examined the individual and combined effects of PTSD diagnosis and MetS on cortical thickness and gray matter volume. Here, we selected individual prefrontal cortical and subcortical gray matter regions-of-interest (ROIs) based on their involvement in the regulation of appetite, satiety, food intake, and reward processing. Secondary objectives included an analysis of the associations of individual MetS components and PTSD illness severity, as assessed by symptoms scores, with brain structure.

First, we hypothesised that there would be brain structural differences between patients with PTSD and TEC. Second, we hypothesised that MetS would be associated with thinner cortical thickness and smaller subcortical gray matter volumes. Third, there would be differential associations of individual MetS components with brain structural measures in PTSD patients compared to controls.

## Materials and methods

### Study design

The present cross-sectional, between-subject design, case-control study was conducted as part of the larger SHARED ROOTS (SR) parent project. The overarching goal of the SR research project was to investigate genomic, neural, cellular and environmental signatures that are common to cardiovascular disease risk and neuropsychiatric disorders and contribute to comorbidity, severity and treatment outcome.

Ethics approval for this research was obtained from the Health Research Ethics Committee at the Faculty of Medicine and Health Sciences of Stellenbosch University (Ethics approval number: HREC N13/08/115). The present study was conducted according to the principles of the Declaration of Helsinki (2013) and the Department of Health Guidelines for Good Clinical Practice (2006). Participants in SR provided voluntary written informed consent. Participants with any health-related concerns on medical examination were referred to health care services.

### Research setting

The present research was conducted at the Faculty of Medicine and Health Sciences, Stellenbosch University. From May 2014 to June 2017, we recruited participants who resided in the northern suburbs of Cape Town and surrounding areas. Several strategies using purposive sampling were selected to recruit and enrol research participants. These approaches included active recruitment at healthcare and community centres, participant identification using existing databases, media advertisements (e.g., print media, radio), and word-of-mouth. Study flyers were also used to recruit patients and controls. These flyers described the symptoms of PTSD but clearly stated that trauma exposed individuals with and without PTSD were being sought for participation in the study. A subset of outpatients with PTSD were also referred for research participation by healthcare workers

### Selection of research participants

**Figure 1.**
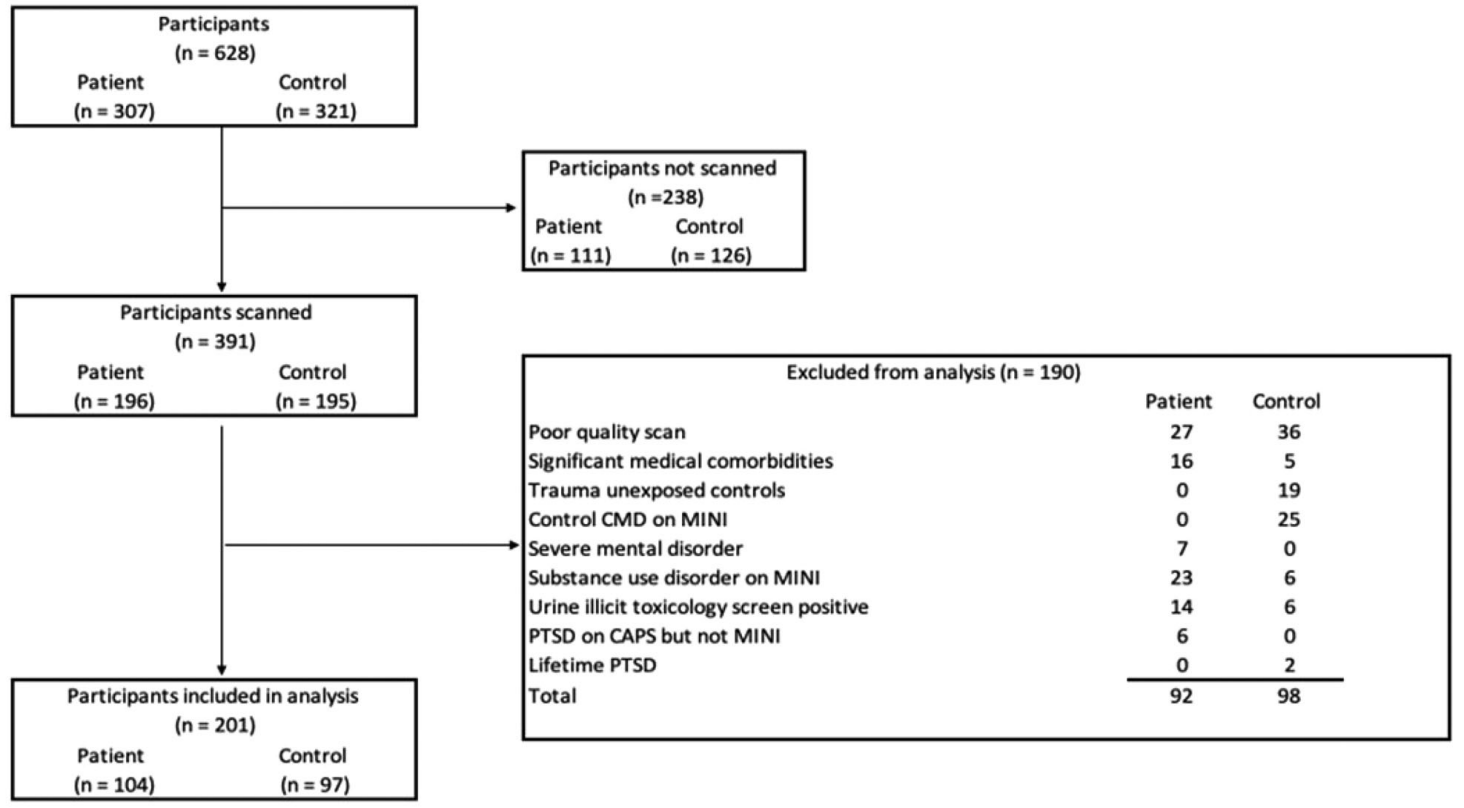
Flow chart illustrating the participant selection procedures used to include suitable cases and controls. Patient = participants with a principal diagnosis of PTSD. CMD: common mental disorder; Mini International Neuropsychiatric Interview; PTSD: posttraumatic stress disorder.

The final sample comprised 104 PTSD cases and 97 TEC (Figure 1). The characteristics of the sample (*n* = 201, 78% female, mean age 41.6 ± 13.1 years) are described in Table 1. We included adults (18 years or older) who provided informed consent and were able to read and write in English or Afrikaans (the predominant languages spoken in this population). For the SHARED ROOTS project, we enrolled individuals of self-reported mixed ancestry. The decision to limit the study to one ethnic group was chiefly to avoid the effects of population stratification on genomic analyses. We included patients who met the Diagnostic and Statistical Manual of Mental Disorders (DSM–5) criteria for PTSD (APA 2013) according to the Clinician Administered Post-traumatic Stress Disorder Scale for DSM–5 (CAPS-5) (Weathers et al. 2018). Sixteen (18%) patients were on treatment with SSRI or TCA antidepressants for a known diagnosis of PTSD prior to screening and enrolment, consistent with the disorder often being underreported and underdiagnosed in our setting (Carey et al. 2003). Three of these patients had also received psychotherapy for PTSD.

**Table 1.** Comparison of socio-demographic, clinical, and biochemical characteristics between patients with PTSD and trauma-exposed controls.

Characteristics	Patients (*n* = 104)	TEC (*n* = 97)	Test statistic	*p*-Value
Age, years, mean (SD)	40.6 (11.8)	42.7 (14.4)	*T* = −1.11	0.268
Sex, female, yes (%)	84 (81%)	73 (75%)	*X*^2^ = 0.44	0.439
Scanner, 3 T Allegra, yes (%)	71 (68%)	57 (60%)	*X*^2^ = 1.57	0.210
Language, *n* (%) Afrikaans English	66 (63%) 38 (37%)	61 (63%) 36 (37%)	*X*^2^ = 0.05	0.942
Secondary schooling completed, yes (%)	31 (30%)	36 (37%)	*X*^2^ = 0.90	0.343
Currently employed, yes (%)	35 (34%)	45 (46%)	*X*^2^ = 2.89	0.089
Alcohol use, last 6 months, yes (%)	44 (42.%)	55 (57%)	*X*^2^ = 3.60	0.058
Tobacco use, last 6 months, yes (%)	42 (40%)	40 (41%)	*X*^2^ = 0.01	0.980
CTQ total scores, mean (SD)	57.0 (22.2)	45.8 (16.7)	*T* = 4.04	<0.001*
LEC-5 total number of lifetime trauma exposures, mean (SD)	8.1 (3.8)	6.0 (3.4)	*T* = 4.12	<0.001*
CAPS-5 total score, mean (SD)	35.7 (8.3)	5.4 (6.6)	*T* = 28.7	<0.001*
MetS criteria met, yes (%)	29 (28%)	27 (28%)	*X*^2^ = 0.01	0.99
Body mass index, mean (SD)	28.0 (6.2)	28.3 (6.8)	*T* = −0.29	0.769
Waist circumference (cm), mean (SD)	88.4 (17.4)	87.7 (14.5)	*T* = 0.31	0.755
Systolic blood pressure (mmHg), mean (SD)	112 (13.2)	116 (15.2)	*T* = −0.26	0.734
Diastolic blood pressure (mmHg), mean (SD)	83 (12.1)	85 (11.1)	*T* = −0.35	0.821
Triglycerides, mmol/L, mean (SD)	1.11 (0.6)	1.15 (0.8)	*T* = −0.38	0.710
Fasting glucose, mmol/L, mean (SD)	5.5 (2.2)	6.1 (2.9)	*T* = −1.68	0.095
HDL cholesterol, mmol/L, mean (SD)	1.39 (0.38)	1.42 (0.38)	*T* = −0.77	0.442

We also enrolled TEC who did not meet the DSM-5 criteria for PTSD. Exclusion criteria for patients and TEC were (1) significant medical comorbidities (e.g., HIV, cancer, cerebrovascular accidents), or severe head injuries, (2) current substance use disorder as determined with the Mini International Neuropsychiatric Interview (MINI) (Sheehan et al. 1998); (3) current serious mental illness (e.g., schizophrenia spectrum disorders, bipolar disorder) as determined based on psychiatric history or the MINI; (4) a substance use disorder on the MINI or positive urine toxicology for methamphetamine, cannabis, or methaqualone; and (5) poor quality MRI scans. In addition, we excluded TEC on treatment for common mental disorders (viz. anxiety and mood disorders) or a lifetime history of PTSD. For the neuroimaging protocol, participants were not scanned if they refused participation, did not arrive for the scan, or had any contraindications which would exclude then from MRI (i.e., had a cardiac pacemaker, metal prosthesis or pin(s), clips on blood vessels, inner ear prosthesis, an infusion pump, a metal intra-uterine contraceptive device, anxiety, claustrophobia, current pregnancy, or were too large to fit the scanner).

### Clinical assessments

Questionnaires were used to capture relevant socio-demographic and clinical data, including information on age, self-reported sex, ethnic background, marital status, employment history, and highest level of schooling completed. Clinical measures of interest included the self-reported use of alcohol, tobacco, and illicit substances (i.e., methamphetamine, cannabis, or methaqualone). Several clinical assessment tools were also used. We examined current and lifetime psychiatric disorders using the MINI. Early life adversity was evaluated using the Childhood Trauma Questionnaire (CTQ) (Bernstein and Fink 1998). The CTQ consists of 28 items, including 25 clinical items and three items on minimalization and denial. Each item is scored on a 5-point Likert scale ranging from 1 (‘never true‘ to 5 (‘very often true‘). A total CTQ severity score is calculated (range, 25–125). In addition, the CTQ allows for the calculation of five childhood trauma subscales for abuse (sexual, physical, emotional) and neglect (emotional, physical).

The diagnosis PTSD was confirmed using the MINI and the CAPS-5 and the severity of PTSD was assessed with the CAPS-5. The Life Events Checklist for DSM-V (LEC-5) (Weathers et al. 2013) was used to screen for lifetime exposure to traumatic events. The LEC-5 examines the personal or indirect experience or witnessing of 16 traumatic events, and includes an additional item used to describe other self-reported stressful events. The index item (worse trauma) is used to assess PTSD severity in the 1 month prior with the CAPS-5. Here, we focused on an overall score calculated based on the total lifetime number of stressful events experienced.

### Metabolic assessments

MetS defines a constellation of cardiovascular risk factors, including obesity, hypertension, dyslipidemia, and dysglycemia (Alberti et al. 2006). Body mass index (BMI) was calculated as body weight in kilograms (kg) divided by height in metres squared (m^2^). Systolic (SBP) and diastolic (DBP) blood pressure were also recorded. A peripheral venous blood sample was collected after an 8–12 hr fasting period for the biochemical determination of fasting glucose, triglyceride, total cholesterol, low-density lipoprotein (LDL), and high-density lipoprotein (HDL) cholesterol levels. The diagnosis of MetS was based on harmonised Joint Interim Statement criteria: (i) increased waist circumference (WC) (men and women ≥90 cm) (Matsha et al. 2013); (ii) elevated triglycerides (>1.7 mmol/l); (iii) low HDL cholesterol (HDL-C) (males <1.0 mmol/l, females <1.3 mmol/l); (iv) SBP ≥130 mmHg or SBP ≥85 mmHg or on antihypertensive drug treatment; and raised fasting glucose ≥5.6 mmol/L or on drug treatment for elevated glucose.

### Structural magnetic resonance imaging

High resolution T1-weighted structural imaging data were acquired on a 3 T Allegra MRI scanner (Erlangen, Germany) (*n* = 128) at the Cape Universities Brain Imaging Centre (CUBIC) for 148 participants, using an updated MEMPRAGE sequence (TR = 2530 ms; TE_1_ = 1.53 ms TE_2_= 3.21, ms, TE_3_ = 4.89 ms, TE_4_ = 6.57 ms, flip-angle: 7 degrees, FoV: 256 mm, 128 slices, 1 isotropic voxel size). A Siemens Skyra scanner (Erlangen, Germany) at the University of Cape Town (UCT) was used to assess an additional 73 participants, using the same MEMPRAGE sequence with the following parameters: TR = 2530 ms, TE_1_ = 1.63 ms, TE_2_= 3.47 ms, TE_3_ = 5.31 ms, TE_4_ = 7.15 ms, flip angle = 7 degrees, FoV = 280 mm, 128 slices, 1 isotropic voxel size.

Scans were processed and analysed using FreeSurfer stable release version 6.0. (http://surfer.nmr.mgh.harvard.edu/). The details of these procedures have been described (Fisch and Dale 2002). For our vertex-based analysis, we used standard automated ROIs generated using the Desikan–Killiany atlas (Desikan et al. 2006). Slices were re-sampled to a three-dimensional image with 1 mm isotropic voxel size. Non-uniform intensity normalisation was performed, with images registered to the Montreal Neurological Institute (MNI) space. A second normalisation step was performed with a different algorithm, with control points automatically identified and normalised to a standard intensity value, followed by automated skull stripping. Gross brain anatomies were delineated into cortical and subcortical labels. Reconstructions were performed with custom batching scripts on the Centre for High Performance Computing (CHPC) (Rosebank, Cape Town) Sun Intel Lengau cluster (http://www.chpc.ac.za/).

### Neuroimaging parameters

Structural brain changes evident in both PTSD and MetS are often bilateral and diffuse, including fronto-temporal cortical thinning and smaller subcortical brain volumes (Song et al. 2015). Nevertheless, there are specific stress-sensitive regions and regions involved in the hedonic regulation of food intake and aberrant reward processing, where similar pathogenic mechanisms may underlie dysregulation in both conditions. For cortical thickness, we selected the following prefrontal and temporal ROIs (*n* = 16): the left and right caudal anterior cingulate, caudal middle frontal, lateral orbito-frontal, medial orbito-frontal, rostral anterior cingulate, rostral middle frontal, superior frontal, and frontal pole cortex. For subcortical gray matter, we selected the left and right ventral diencephalon (VD), hippocampus, amygdala, and nucleus accumbens (*n* = 8). The VD is comprised of several structures, which are closely related to autonomic regulation, including appetite and satiety, consistent with the physiological regulation of food intake. These include the hypothalamus, substantia nigra, red nucleus, mamillary bodies, and the geniculate nucleus . These form part of a proposed ‘core eating network‘ (Chen et al. 2015), and are closely positioned and function at the interface between metabolic and limbic regulation of food intake, salience, appetite, and reward. Hypothalamic dysconnectivity to fronto-limbic structures is implicated in the pathogenesis of both obesity and PTSD (Berthoud et al. 2017). Obesity is further characterised by decreased connectivity between the prefrontal cortex and subcortical structures, including the hippocampus and amygdala (Donofry et al. 2022). Smaller volumes for these subregions have also been associated with both PTSD severity and MetS (Farr et al. 2014).

We selected pre-defined *a priori* ROIs to limit the number of comparisons. We also sought to determine whether the effects evident for these ROIs were indeed localised. Global cortical thickness and total subcortical gray matter volumes were therefore selected as control parameters, and covaried for as appropriate in our ROI analyses.

### Statistical analysis

Statistical analyses were performed using R Studio software (version 20). Categorical data were described as counts and percentages (%) and compared between PTSD cases and controls using a Chi-squared or Fisher’s exact test, as appropriate. Visual inspection of numerical data was performed to check for skewness, kurtosis, and lordosis of numerical variables. Continuous numerical data were presented as the mean with standard deviation (SD) and compared between patient groups using a Student’s *t*-test.

For our primary objective: we constructed two multivariate, multiple hierarchical regression models, one for cortical thickness, and one for subcortical gray matter, across the individual ROIs (prefrontal cortical ROI: *n* = 16; subcortical gray matter; *n* = 8). For both multivariate models, in block I, we entered socio-demographic predictors; in block II, we entered PTSD diagnosis; in block III, we added MetS status; and in block IV, we included the interaction of PTSD diagnosis and MetS status. Post-hoc linear regression models were run to discern the effects of PTSD diagnosis and MetS on individual ROIs. We adjusted for age, sex, scanner site, alcohol, and tobacco use in our initial models. In addition, we adjusted for global cortical thickness when examining the prefrontal cortical ROIs, in order to determine whether the effects of PTSD diagnosis vs. MetS status were indeed region-specific. The subcortical gray matter volume ROIs were proportion-adjusted for ICV, and expressed as the %eTIV.

For our secondary objectives, informed by our main findings, we ran linear regression to explore the effects of individual MetS variables and PTSD severity on the brain of individual ROIs identified as significant in our primary analyses. The ROIs were the dependent variables, with individual MetS components (i.e., waist circumference, fasting glucose, SBP, DBP, triglycerides, and HDL cholesterol levels) and PTSD severity (i.e., CAPS-5 scores) as predictors, adjusting for age, sex, scanner sequence, tobacco and alcohol use. CAPS-5 scores and all individual MetS features were entered into the same model with individuals ROI as dependent variables. CTQ total score and LEC total score were also controlled for as covariates. Missing data items were very few and excluded listwise from the analysis. Statistical analyses were performed using R Studio software. A *p*-value <0.05 was considered statistically significant at the unadjusted level for our main multivariate models. In our post-hoc primary linear regressions, we adjusted for multiple comparisons using the Benjamini-Hochberg method (Benjamini and Hochberg 1995).

## Results

### Clinical, metabolic, and imaging data compared between cases and controls

The sample included 104 patients with PTSD and 97 TEC. The two groups were balanced in terms of age, sex, secondary schooling completed, current employment over the last 12 months, marital status, alcohol, and tobacco use. MetS prevalence was similar in patients with PTSD (*n* = 29; 28%) and controls (*n* = 27; 28%) (*p* > 0.05). CAPS-5, CTQ and LEC scores were significantly higher in patients than controls (*p* < 0.001). The characteristics of the sample are presented in Table 1. Box-and-whisker plots of CTQ total score and LEC total scores, compared between cases and controls, are shown in Supplemental Figures 2A and B, respectively.

### PTSD diagnosis and metabolic syndrome effects on prefrontal cortical thickness

Hierarchical multivariate linear regression was used to model the effects of PTSD diagnosis and MetS status on prefrontal cortical thickness ROIs. In block I, PTSD diagnosis had no significant effect (F(1,181) = 1.09, *p* = 0.372), while in block II, MetS had a significant effect across the grouped prefrontal cortical ROIs (F(1,181) = 2.47, *p* = 0.003). Addition of MetS status improved the performance of the model. In block III, the interaction effect of PTSD diagnosis X MetS was not significant. Results are summarised in Supplemental Table S1.

Post-hoc regressions for individual cortical thickness ROIs showed associations of both PTSD diagnosis and MetS status with thinner prefrontal cortical thickness across several ROIs at the unadjusted level (*p* < 0.05). Following correction for multiple testing (*p* = 0.0032), PTSD and MetS were both associated with thinner right caudal middle frontal cortices, adjusting for age, sex, scanner sequence, alcohol and tobacco use, and global cortical thickness (Supplemental Figure S2). Independent of PTSD diagnosis, MetS was associated with thinner left and right superior frontal as well as right caudal anterior cingulate cortex, adjusting for age, sex, scanner sequence, alcohol and tobacco use, and global cortical thickness. These results are summarised in Supplemental Table S2.

### Association of PTSD severity and individual metabolic syndrome components with prefrontal cortical thickness

Informed by our main analyses, we conducted follow-up linear regressions to model the effects of PTSD severity and the individual MetS components on prefrontal cortical thickness ROIs that were significantly associated with MetS in our initial post-hoc regression models. We found that increased triglycerides predicted thinner left and right superior frontal and right caudal middle frontal cortex, while higher waist circumference predicted thinner right superior frontal and right caudal middle frontal cortex, adjusting for age, sex, CAPS-5 scores, the total number of lifetime traumas exposed to (LEC), CTQ total scores, alcohol and tobacco use, scanner sequence, and other MetS components. Linear regression outputs are summarised in Supplemental Table S3.

### Effects of PTSD diagnosis and metabolic syndrome on subcortical gray matter volumes

Hierarchical multivariate linear regression was used to model the effects of PTSD diagnosis and MetS status across the grouped subcortical gray matter ROIs. In block I, PTSD diagnosis had a significant effect (F(1,185) = 1.88, *p* = 0.049), while in block II, both PTSD diagnosis (F(1,184) = 2.38, *p* = 0.011) and MetS (F(1,184) = 2.39, *p* = 0.011) had significant and independent effects, and addition of MetS status improved the performance of the model. There was however no significant interactive effect between PTSD diagnosis and MetS across subcortical gray matter ROIs (F(1,183) = 0.04, *p* = 0.570). These results are summarised in Supplemental Table S4.

Post-hoc regression models showed that, following correction for multiple testing (*p* = 0.0059), PTSD diagnosis and MetS had independent effects on left VD volumes, adjusting for age, sex, scanner sequence, alcohol, and tobacco use (Supplemental Figure S3). There were no other significant associations of either PTSD diagnosis or MetS on other subcortical gray matter ROIs at the adjusted significance level. These results are summarised in Supplemental Table S5.

### Associations of PTSD severity and individual metabolic syndrome components with subcortical gray matter volumes

We conducted follow-up linear regressions to examine the effects PTSD severity and the individual MetS components on left and right VD volumes. We found that higher mean CAPS-5 total scores and increased fasting glucose levels predicted smaller left VD volumes, adjusting for age, sex, LEC and CTQ scores, alcohol and tobacco use, scanner site and sequence, waist circumference, systolic and diastolic blood pressure, triglycerides, and HDL cholesterol levels. Linear regression outputs are summarised in Supplemental Table S6.

## Discussion

Several key findings deserve mention. First, MetS status was an independent predictor of thinner prefrontal cortical thickness across multiple ROIs, adjusting for age, sex, alcohol and nicotine use, and scanner site and sequence. In contrast, both PTSD diagnosis and MetS predicted thinner subcortical gray matter volumes across the grouped ROIs, adjusting for the same confounders. Post-hoc linear regressions showed associations of individual MetS components with thinner cortical thickness across several ROIs, which survived correction for multiple testing. In particular, our results suggest that PTSD diagnosis and MetS exert independent effects on brain structure. Furthermore, our results support the differential associations of PTSD severity and individual MetS features with brain structural differences.

Our findings are also consistent with previously documented associations of MetS with frontal cortical thinning and subcortical volume loss in the general population (Song et al. 2015; Gómez-Apo et al. 2021). Our research adds to existing research showing putative differential effects of PTSD diagnosis and MetS on brain structure (Wolf et al. 2016). In addition, putative differential associations of the individual MetS components with cortical thickness and subcortical gray matter volumes were found. Importantly, our research suggests independent, rather than additive, associations of PTSD and MetS with brain structure.

Second, MetS was associated with thinner prefrontal cortical thickness across multiple ROI. This finding is consistent with frontal cortical thinning described for MetS (Gómez-Apo et al. 2021). For example, Medic et al. (2016) found that increased BMI was associated with a thinner prefrontal cortex across several ROIs in healthy volunteers (*n* = 202). Similar to our results, Hassenstab et al. (2012) described thinner ACC thickness in obese vs. non-obese individuals. Involvement of the superior frontal and caudal middle frontal gyrus in obesity is well-known (Marqués-Iturria et al. 2013; Franz et al. 2019; Opel et al. 2021). Our findings are similar to Wolf et al. (2016), who, in a sample of PTSD cases and controls, found that MetS predicted thinner cortical thickness across multiple frontal cortical regions, including the anterior cingulate cortex, adjusting for PTSD diagnosis. However, in the Wolf et al. (2016) study, PTSD diagnosis, in turn, predicted MetS. In contrast, the prevalence of MetS was balanced between cases and controls in our sample. There are several expected reasons for this. We purposively balanced cases and controls for MetS prevalence at enrolment in the current study. Our sample was also drawn from a population where a high overall prevalence of obesity has been described (Erasmus et al. 2012). MetS was thus not a PTSD-specific predictor of brain structural differences.

Third, a region-specific effect for MetS on left VD volumes, correcting for multiple testing, was evident. In keeping with the literature, associations between obesity and smaller VD volumes have also been described (Marqués-Iturria et al. 2013). The VD is comprised of different structures, but mainly includes the hypothalamus, which is a known master orchestrator of food intake, and is associated with obesity risk (Curran et al. 2013). The hypothalamus is connected to multiple limbic structures involved in reward anticipation and emotion processing which are in turn subject to inhibitory control from the dorso-lateral prefrontal and anterior cingulate cortices (Roger et al. 2021).

Fourth, we found evidence for differential associations of some of the individual MetS components with prefrontal cortical thickness (triglycerides, HDL cholesterol) and subcortical gray matter ROI (fasting glucose). Several earlier studies have described the contribution of some MetS components, but not others, to thinner cortical thickness in population-based studies (Leritz et al. 2011). For example, Bahchevanov et al. (2018) found that increased WC, but not the other MetS features, predicted thinner cortical thickness in the general population, which is consistent with our findings. In a similar fashion, differential associations of abnormal lipid profiles, but not obesity, have been associated with cognitive performance in adolescents (Bugge et al. 2018).

The neurobiological mechanisms whereby PTSD and MetS contribute to cortical thinning and subcortical volume loss show some extent of overlap. These include inflammation, oxidative stress, immune dysregulation, altered cortisol metabolism, epigenetic alterations and impaired blood-brain barrier permeability (Kibler et al. 2014; Levine et al. 2014). In patients with PTSD, altered cortisol metabolism causes impaired HPA axis regulation (Yehuda et al. 1995), contributing to systemic inflammation that is a documented mechanism implicated in weight gain (Levine et al. 2014). The lack of a main association between PTSD diagnosis and prefrontal cortical thickness was thus unexpected, given that most, albeit not all, studies have described this effect (Karl et al. 2006). Nevertheless, consistent with this evidence, we found that PTSD diagnosis and CAPS-5 scores predicted thinner right caudal middle frontal cortex thickness, which was also associated with WC in our secondary regression models. Our findings, taken together, support an association between MetS features and loco-regional cortical thickness differences, largely independent of PTSD, which was however associated with right caudal middle frontal cortical thickness.

Overall, our results are consistent with the overall hypothesis that grounded our research project, i.e., that both PTSD diagnosis and MetS are associated with similar brain structural differences. Our findings also implicate MetS diagnosis, but not PTSD diagnosis, as an independent predictor of smaller subcortical gray matter volumes. Our research conducted in the schizophrenia cohort of the ‘SHARED ROOTS‘ study similarly found that increased BMI predicted thinner prefrontal cortical thickness, independent of schizophrenia diagnosis.

Our study has several limitations, including a cross-sectional design, which limits our ability to infer the causal nature of a relationship between PTSD, MetS, and brain structure. Our ROI analysis, while including key prefrontal and cortico-limbic structures, was not inclusive of all brain changes described for MetS, e.g., decreased cortical thickness for the temporal poles, fusiform gyrus, and insula (Wolf et al. 2016). Importantly, structural brain changes might also precede the onset of cortical thickness changes in MetS and PTSD. Second, our results cannot necessarily be extrapolated to other PTSD samples. Replication and longitudinal studies will be required to address these concerns. Third, since we could not assess patients who were too large to fit into the scanner, selection bias might have been present and limited our ability to assess severe and morbid obesity in relation to PTSD symptoms and brain structure. Fourth, more research is needed to understand the effects of dietary and lifestyle factors on the relationships between PTSD, MetS, and brain structure. Lastly, structural MRI measures may be confounded by many epiphenomena and artefacts and observed differences do not necessarily imply abnormalities. However, our research also has important strengths, including a large sample size, rigorous, standardised clinical assessments of both PTSD and MetS, and evaluation of relevant structural ROIs linked to hedonic and homeostatic regulation of food intake. We also assessed and controlled for important confounding factors, including age, sex, alcohol and nicotine use, childhood trauma exposure, and lifetime exposure to trauma.

In conclusion, our research supports associations of MetS with thinner prefrontal cortical thickness in a mixed ancestry population from South Africa. We also demonstrated the effects of both PTSD diagnosis and MetS as independent predictors of smaller subcortical gray matter volumes. Our results provide an important foundation for multi-site studies utilising multi-modal imaging to further explore the longitudinal associations of PTSD and MetS with brain structural changes. Clinicians should be cognization of MetS changes and their sequalae in patients with PTSD. Clinical monitoring and timely intervention might help prevent the development of MetS-associated complications.

## Supplementary Material

Supplemental Material

Supplemental Material

Supplemental Material

Supplemental Material

## Data Availability

Data pertaining to this study are being used to address additional aims and cannot be shared publicly at this stage. The study collaborative team will review individual requests to access data.
