# Supplementary material for: Independent effects of posttraumatic stress disorder diagnosis and metabolic syndrome status on prefrontal cortical thickness and subcortical gray matter volumes
Source: Dialogues Clin Neurosci. 2023 Jul 27;25(1):64–74. doi: 10.1080/19585969.2023.2237525 (PMC10375918; doi:10.1080/19585969.2023.2237525)
Supplement: Supplemental Material [file TDCN_A_2237525_SM7138.docx]

Supplemental Table S1. Hierarchical multivariate multiple regression outputs modelling the effects of posttraumatic stress disorder (PTSD) diagnosis (block I), metabolic syndrome (MetS) status (block II), and PTSD diagnosis X MetS interaction (block III) on prefrontal cortical thickness ROIs. All models were adjusted for age, sex, scanner sequence, alcohol, and nicotine use, and global cortical thickness.

| Predictor | F-value | Df | P-value |
| --- | --- | --- | --- |
| Block I (Model 1) | | | |
| PTSD | 1.09 | 1,181 | 0.372 |
| Block II (Model 2) | | | |
| PTSD | 1.12 | 1,180 | 0.346 |
| MetS | 2.47 | 1,180 | 0.003* |
| Block III (Model 3) | | | |
| PTSD | 1.11 | 1,179 | 0.348 |
| MetS | 2.46 | 1,179 | 0.003* |
| PTSD X MetS | 0.33 | 1,179 | 0.989 |

|  | Df | Pillai | F-value (df) | P-value |
| --- | --- | --- | --- | --- |
| Model 1 | - | - | - | - |
| Model 2 | 1,193 | 0.16 | 2.46 | 0.003* |
| Model 3 | 1,192 | 0.03 | 0.33 | 0.989 |

Supplemental Table S2. Hierarchical linear regression model showing the effects of posttraumatic stress disorder diagnosis (block I) and metabolic syndrome (block II) as well as their interactions (block III) on prefrontal cortical thickness ROIs. All models were adjusted for age, sex, scanner sequence, alcohol and nicotine use, and global cortical thickness.

|  | Block I | | | Block II | | | Block III | | |
| --- | --- | --- | --- | --- | --- | --- | --- | --- | --- |
| Predictor | Beta | T-value | P-value | Beta | T-value | P-value | Beta | T-value | P-value |
| Left frontal pole cortex | | | | | | | | | |
| PTSD | -0.02 | -0.69 | 0.492 | -0.02 | -0.69 | 0.494 | -0.03 | -0.83 | 0.409 |
| MetS | - | - | - | 0.01 | 0.18 | 0.290 | -0.05 | -0.69 | 0.492 |
| PTSD x MetS | - | - | - | - | - | - |  | 0.47 | 0.638 |
| R^2^ | 0.361 | | | 0.361 | | | 0.362 | | |
| ΔR^2^ |  | | | 0.000 | | | 0.001 | | |
| Right frontal pole cortex | | | | | | | | | |
| PTSD | 0.01 | 0.15 | 0.885 | 0.01 | 0.14 | 0.889 | -0.03 | 0.24 | 0.812 |
| MetS | - | - | - | -0.03 | -0.45 | 0.588 | -0.05 | 0.04 | 0.966 |
| PTSD x MetS | - | - | - | - | - | - | -0.02 | -0.23 | 0.816 |
| R^2^ | 0.256 | | | 0.257 | | | 0.258 | | |
| ΔR^2^ |  | | | 0.001 | | | 0.001 | | |
| Left superior frontal cortex | | | | | | | | | |
| PTSD | -0.02 | -1.41 | 0.161 | -0.02 | -1.47 | 0.143 | -0.02 | -1.20 | 0.233 |
| **MetS** | **-** | **-** | **-** | **-0.06** | **-3.35** | **0.001*** | -0.05 | -0.97 | 0.332 |
| PTSD x MetS | - | - | - | - | - | - | -0.01 | -0.04 | 0.899 |
| R^2^ | 0.720 | | | 0.735 | | | 0.735 | | |
| ΔR^2^ |  | | | 0.033 [A] | | | 0.000 | | |
| Right superior frontal cortex | | | | | | | | | |
| PTSD | -0.02 | -0.94 | 0.349 | -0.02 | -0.96 | 0.337 | -0.016 | -0.89 | 0.376 |
| **MetS** | **-** | **-** | **-** | **-0.08** | **-4.41** | **<0.001**** | -0.08 | -1.46 | 0.145 |
| PTSD x MetS | - | - | - | - | - | - | 0.01 | 0.03 | 0.978 |
| R^2^ | 0.691 | | | 0.712 | | | 0.719 | | |
| ΔR^2^ |  | | | 0.021 [A] | | | 0.007 | | |
| Left caudal middle frontal cortex | | | | | | | | | |
| PTSD | -0.03 | -1.71 | 0.089 | -0.03 | -1.70 | 0.091 | -0.03 | -1.79 | 0.071 |
| MetS | - | - | - | 0.01 | 0.35 | 0.730 | -0.06 | -0.50 | 0.616 |
| PTSD x MetS | - | - | - | - | - | - | 0.003 | 0.67 | 0.512 |
| R^2^ | 0.557 | | | 0.557 | | | 0.558 | | |
| ΔR^2^ |  | | | <0.001 | | | 0.001 | | |
| Right caudal middle frontal cortex | | | | | | | | | |
| PTSD | -0.05 | -2.81 | 0.005* | **-0.05** | **-2.92** | **0.003**** | -0.06 | -2.88 | 0.004* |
| **MetS** | **-** | **-** | **-** | **-0.06** | **-2.88** | **0.003**** | -0.10 | -2.65 | 0.100 |
| PTSD x MetS | - | - | - | - | - | - | 0.03 | 0.73 | 0.960 |
| R^2^ | 0.575 | | | 0.593 | | | 0.221 | | |
| ΔR^2^ |  | | | 0.018 [A] | | | 0.00 | | |
| Left rostral middle frontal cortex | | | | | | | | | |
| PTSD | -0.02 | -1.43 | 0.156 | -0.02 | -1.44 | 0.151 | -0.03 | -1.73 | 0.087 |
| MetS | - | - | - | -0.01 | -0.78 | 0.435 | -0.05 | -1.16 | 0.249 |
| PTSD x MetS | - | - | - | - | - | - | 0.03 | 0.95 | 0.344 |
| R^2^ | 0.612 | | | 0.613 | | | 0.615 | | |
| ΔR^2^ |  | | | 0.001 | | | 0.002 | | |
| Right rostral middle frontal cortex | | | | | | | | | |
| PTSD | -0.02 | -1.32 | 0.187 | -0.02 | -1.17 | 0.173 | -0.01 | -0.56 | 0.579 |
| MetS | - | - | - | -0.03 | -1.78 | 0.076 | 0.03 | 0.54 | 0.588 |
| PTSD x MetS | - | - | - | - | - | - | -0.04 | -1.23 | 0.226 |
| R^2^ | 0.563 | | | 0.570 | | | 0.573 | | |
| ΔR^2^ |  | | | 0.007 | | | 0.003 | | |
| Left caudal anterior cingulate cortex | | | | | | | | | |
| PTSD | -0.04 | -1.81 | 0.239 | -0.04 | -1.17 | 0.239 | -0.01 | -0.56 | 0.579 |
| MetS | - | - | - | -0.01 | -0.15 | 0.878 | 0.03 | 0.54 | 0.588 |
| PTSD x MetS | - | - | - | - | - | - | -0.04 | -1.23 | 0.226 |
| R^2^ | 0.048 | | | 0.048 | | | 0.048 | | |
| ΔR^2^ |  | | | <0.001 | | | <0.001 | | |
| Right caudal anterior cingulate cortex | | | | | | | | | |
| PTSD | 0.012 | 0.65 | 0.514 | 0.02 | 0.61 | 0.542 | 0.03 | 0.98 | 0.330 |
| MetS | - | - | - | **-0.09** | **-2.96** | **0.003**** | <0.01 | -0.01 | 0.588 |
| PTSD x MetS | - | - | - | - | - | - | -0.06 | -0.89 | 0.377 |
| R^2^ | 0.065 | | | 0.094 | | | 0.573 | | |
| ΔR^2^ |  | | | 0.029 [A] | | | 0.003 | | |
| Left rostral anterior cingulate cortex | | | | | | | | | |
| PTSD | 0.01 | -0.41 | 0.685 | 0.01 | 0.41 | 0.684 | 0.03 | 0.56 | 0.519 |
| MetS | - | - | - | <0.01 | 0.09 | 0.926 | 0.07 | 0.58 | 0.564 |
| PTSD x MetS | - | - | - | - | - | - | -0.05 | -0.58 | 0.562 |
| R^2^ | 0.190 | | | 0.190 | | | 0.192 | | |
| ΔR^2^ |  | | | <0.001 | | | 0.002 | | |
| Right rostral anterior cingulate cortex | | | | | | | | | |
| PTSD | 0.03 | 0.96 | 0.339 | 0.03 | 0.96 | 0.338 | 0.04 | 1.02 | 0.519 |
| MetS | - | - | - | 0.01 | 0.20 | 0.843 | 0.05 | 0.42 | 0.564 |
| PTSD x MetS | - | - | - | - | - | - | -0.07 | -0.38 | 0.707 |
| R^2^ | 0.109 | | | 0.109 | | | 0.110 | | |
| ΔR^2^ |  | | | <0.001 | | | 0.001 | | |
| Left medial orbitofrontal cortex | | | | | | | | | |
| PTSD | -0.001 | -0.05 | 0.963 | -0.001 | -0.06 | 0.952 | 0.01 | 0.45 | 0.656 |
| MetS | - | - | - | -0.02 | -0.69 | 0.493 | 0.06 | 0.68 | 0.496 |
| PTSD x MetS | - | - | - | - | - | - | -0.05 | -0.97 | 0.333 |
| R^2^ | 0.296 | | | 0.298 | | | 0.302 | | |
| ΔR^2^ |  | | | 0.002 | | | 0.004 | | |
| Right medial orbitofrontal cortex | | | | | | | | | |
| PTSD | 0.002 | 0.60 | 0.545 | 0.01 | 0.56 | 0.575 | 0.01 | 0.94 | 0.349 |
| MetS | - | - | - | -0.02 | -2.26 | 0.026* | 0.06 | 0.08 | 0.936 |
| PTSD x MetS | - | - | - | - | - | - | -0.05 | -0.89 | 0.372 |
| R^2^ | 0.374 | | | 0.390 | | | 0.393 | | |
| ΔR^2^ |  | | | 0.016 [A] | | | 0.003 | | |
| Left lateral orbitofrontal cortex | | | | | | | | | |
| PTSD | <0.001 | 0.03 | 0.980 | 0.01 | 0.05 | 0.962 | -0.01 | -0.41 | 0.680 |
| MetS | - | - | - | 0.02 | 0.08 | 0.282 | -0.03 | -0.47 | 0.634 |
| PTSD x MetS | - | - | - | - | - | - | 0.03 | 0.88 | 0.376 |
| R^2^ | 0.544 | | | 0.547 | | | 0.549 | | |
| ΔR^2^ |  | | | 0.003 | | | 0.002 | | |
| Right lateral orbitofrontal cortex | | | | | | | | | |
| PTSD | 0.01 | 0.28 | 0.781 | 0.01 | 0.28 | 0.774 | -0.01 | 0.28 | 0.778 |
| MetS | - | - | - | 0.01 | 0.45 | 0.653 | -0.03 | 0.22 | 0.827 |
| PTSD x MetS | - | - | - | - | - | - | -0.003 | -0.07 | 0.945 |
| R^2^ | 0.430 | | | 0.430 | | | 0.430 | | |
| ΔR^2^ |  | | | <0.001 | | | <0.001 | | |

PTSD = post-traumatic stress disorder; MetS = metabolic syndrome

A= addition of MetS improved the prediction capabilities of the model beyond PTSD diagnosis in the final model.

*indicates statistical significance at the unadjusted level; **indicates statistical significance following post-hoc correction for multiple testing (p-adjusted threshold= 0.0031) for the final model.

Supplemental Table S3. Effects of individual metabolic syndrome components on left and right superior frontal and right caudal middle frontal cortical thickness. All models were adjusted for age, sex, scanner sequence, alcohol and nicotine use, CTQ and LEC-5 total scores.

| DV = Left superior frontal cortex  F (9, 191) = 24.5, R^2^ = 0.54, p <0.001 | | | |
| --- | --- | --- | --- |
| Predictor | Beta-coefficient | T-value | P-value |
| CAPS-5 total scores | 0.001 | 1.49 | 0.139 |
| Waist circumference | <0.001 | -1.78 | 0.089 |
| Fasting glucose | -0.004 | -1.08 | 0.390 |
| Systolic blood pressure | -0.001 | -0.63 | 0.294 |
| Diastolic blood pressure | <0.001 | 0.20 | 0.856 |
| **Triglycerides** | **-0.07** | **-4.05** | **<0.001*** |
| High-density lipoprotein cholesterol | -0.07 | -3.36 | 0.051 |
| DV = Right superior frontal cortex  F (9, 191) = 21.4, R^2^ = 0.51, p <0.001 |  |  |  |
| Predictor | Beta-coefficient | T-value | P-value |
| CAPS-5 total scores | 0.001 | 1.21 | 0.192 |
| **Waist circumference** | **-0.002** | **-2.21** | **0.025*** |
| Fasting glucose | -0.002 | -0.64 | 0.481 |
| Systolic blood pressure | -0.001 | -1.48 | 0.165 |
| Diastolic blood pressure | <0.001 | 0.20 | 0.562 |
| **Triglycerides** | **-0.06** | **-2.88** | **0.004*** |
| High-density lipoprotein cholesterol | -0.07 | -1.91 | 0.051 |
| DV = Right caudal middle frontal cortex  F (9, 191) = 12.7, R^2^ = 0.37, p <0.001 |  |  |  |
| Predictor | Beta-coefficient | T-value | P-value |
| **CAPS-5 total scores** | **0.002** | **2.94** | **0.001*** |
| **Waist circumference** | **-0.001** | **-2.31** | **0.022*** |
| Fasting glucose | -0.001 | -0.05 | 0.967 |
| Systolic blood pressure | -0.002 | -1.42 | 0.158 |
| Diastolic blood pressure | <0.001 | 0.15 | 0.563 |
| **Triglycerides** | **-0.06** | **-3.05** | **<0.001*** |
| High-density lipoprotein cholesterol | -0.03 | -1.09 | 0.277 |

DV = dependent variable

Supplemental Table S4. Hierarchical multivariate multiple regression outputs modelling the effects of PTSD diagnosis (block I), MetS (block II), and the PTSD diagnosis X MetS interaction (block III) across the subcortical gray matter regions-of-interest. All models were adjusted for age, sex, scanner sequence, alcohol and nicotine use, and ICV.

| Predictor | F-value | df | p-value |
| --- | --- | --- | --- |
| Block I (Model 1) | | | |
| PTSD | 1.88 | 1,185 | 0.049* |
| Block II (Model 2) | | | |
| PTSD | 2.38 | 1,184 | 0.011* |
| MetS | 2.39 | 1,184 | 0.011* |
| Block III (Model 3) | | | |
| PTSD | 0.11 | 1,183 | 0.012* |
| MetS | 0.12 | 1,183 | 0.011* |
| PTSD X MetS | 0.04 | 1,183 | 0.570 |

|  | df | Pillai | F-value (df) | P-value |
| --- | --- | --- | --- | --- |
| Model 1 | - | - | - | - |
| Model 2 | 1,217 | 0.08 | 1,92 | 0.043* |
| Model 3 | 1,217 | 0.02 | 0.41 | 0.942 |

Supplemental Table S5. Hierarchical linear regression model showing the effects of posttraumatic stress disorder diagnosis (block I) and metabolic syndrome (block II) as well as their interactions (block III) on the subcortical gray matter ROIs. All models were adjusted for age, sex, scanner sequence, alcohol and nicotine use, and ICV.

| \|  \| Block I \| \| \| \| \| \| Block II \| \| \| \| \| \| \| Block III \| \| \| \| \| \| --- \| --- \| --- \| --- \| --- \| --- \| --- \| --- \| --- \| --- \| --- \| --- \| --- \| --- \| --- \| --- \| --- \| --- \| --- \| \| Predictor \| Beta \| \| T-value \| \| P-value \| \| Beta \| \| T-value \| \| P-value \| \| \| Beta \| T-value \| \| P-value \| \| \| Left ventral diencephalon \| \| \| \| \| \| \| \| \| \| \| \| \| \| \| \| \| \| \| \| **PTSD** \| \| **-0.01** \| \| **-3.51** \| \| **0.001*** \| \| **-0.01** \| \| **-3.03** \| \| **0.002*** \| **-0.01** \| \| \| **-2.88** \| \| **0.004*** \| \| **MetS** \| \| **-** \| \| **-** \| \| **-** \| \| **-0.01** \| \| **-2.48** \| \| **0.003*** \| -0.02 \| \| \| -1.26 \| \| 0.209 \| \| PTSD x MetS \| \| - \| \| - \| \| - \| \| - \| \| - \| \| - \| 0.004 \| \| \| 0.47 \| \| 0.642 \| \| R^2^ \| \| 0.149 \| \| \| \| \| \| 0.233 \| \| \| \| \| 0.234 \| \| \| \| \| \| \| ΔR^2^ \| \|  \| \| \| \| \| \| 0.084 [A] \| \| \| \| \| 0.001 \| \| \| \| \| \|   Right ventral diencephalon | | | | | | | | | | |
| --- | --- | --- | --- | --- | --- | --- | --- | --- | --- | --- | --- | --- | --- | --- | --- | --- | --- | --- | --- | --- | --- | --- | --- | --- | --- | --- | --- | --- | --- | --- | --- | --- | --- | --- | --- | --- | --- | --- | --- | --- | --- | --- | --- | --- | --- | --- | --- | --- | --- | --- | --- | --- | --- | --- | --- | --- | --- | --- | --- | --- | --- | --- | --- | --- | --- | --- | --- | --- | --- | --- | --- | --- | --- | --- | --- | --- | --- | --- | --- | --- | --- | --- | --- | --- | --- | --- | --- | --- | --- | --- | --- | --- | --- | --- | --- | --- | --- | --- | --- | --- | --- | --- | --- | --- | --- | --- | --- | --- | --- | --- | --- | --- | --- | --- | --- | --- | --- | --- | --- | --- | --- | --- | --- | --- | --- | --- | --- | --- | --- | --- | --- | --- | --- | --- | --- | --- | --- | --- | --- | --- | --- | --- | --- | --- | --- | --- | --- | --- | --- | --- | --- | --- | --- | --- | --- | --- | --- | --- | --- | --- | --- | --- |
| PTSD | -0.008 | -2.15 | 0.033* | -0.009 | -2.34 | 0.026* | -0.009 | -2.17 | 0.031* | |
| MetS | - | - | - | -0.01 | -2.49 | 0.014* | -0.02 | -1.24 | 0.218 | |
| PTSD x MetS | - | - | - | - | - | - | -0.003 | 0.438 | 0.663 | |
| R^2^ | 0.160 | | | 0.186 | | | 0.187 | | | |
| ΔR^2^ |  | | | 0.026 | | | 0.001 | | | |
| Left hippocampus | | | | | | | | | |  |
| PTSD | -0.001 | -0.07 | 0.948 | -0.001 | -0.07 | 0.941 | -0.001 | 0.10 | 0.917 |  |
| MetS | - | - | - | -0.001 | -0.35 | 0.730 | -0.001 | 0.22 | 0.824 |  |
| PTSD x MetS | - | - | - | - | - | - | -0.003 | 0.47 | 0.642 |  |
| R^2^ | 0.122 | | | 0.123 | | | 0.234 | | |  |
| ΔR^2^ |  | | | 0.001 | | | 0.001 | | |  |

| Right hippocampus | | | | | | | | | |
| --- | --- | --- | --- | --- | --- | --- | --- | --- | --- |
| PTSD | 0.001 | 0.16 | 0.872 | 0.001 | 0.14 | 0.890 | 0.001 | 0.33 | 0.744 |
| MetS | - | - | - | -0.001 | -0.95 | 0.345 | -0.001 | 0.10 | 0.923 |
| PTSD x MetS | - | - | - | - | - | - | -0.003 | -0.43 | 0.665 |
| R^2^ | 0.119 | | | 0.123 | | | 0.234 | | |
| ΔR^2^ |  | | | 0.004 | | | 0.001 | | |

| Left amygdala | | | | | | | | | |
| --- | --- | --- | --- | --- | --- | --- | --- | --- | --- |
| PTSD | -0.001 | -0.60 | 0.549 | -0.001 | -0.64 | 0.524 | 0.001 | -1,25 | 0.212 |
| MetS | - | - | - | -0.003 | -1,52 | 0.129 | -0.001 | -1,87 | 0.063 |
| PTSD x MetS | - | - | - | - | - | - | 0.006 | 1.44 | 0.151 |
| R^2^ | 0.040 | | | 0.052 | | | 0.062 | | |
| ΔR^2^ |  | | | 0.012 | | | 0.010 | | |

| Right amygdala | | | | | | | | | |
| --- | --- | --- | --- | --- | --- | --- | --- | --- | --- |
| PTSD | -0.002 | -0.86 | 0.372 | -0.001 | -0.86 | 0.392 | -0.003 | -1.30 | 0.192 |
| MetS | - | - | - | -0.003 | 0.99 | 0.321 | -0.005 | -0.77 | 0.444 |
| PTSD x MetS | - | - | - | - | - | - | 0.006 | 1.18 | 0.238 |
| R^2^ | 0.039 | | | 0.043 | | | 0.050 | | |
| ΔR^2^ |  | | | 0.004 | | | 0.007 | | |

| Left nucleus accumbens | | | | | | | | | |
| --- | --- | --- | --- | --- | --- | --- | --- | --- | --- |
| PTSD | -0.001 | -0.42 | 0.676 | -0.001 | -0.83 | 0.706 | -0.002 | -0.48 | 0.634 |
| MetS | - | - | - | -0.002 | -1.31 | 0.193 | -0.001 | -1.26 | 0.416 |
| PTSD x MetS | - | - | - | - | - | - | 0.004 | 0.47 | 0.685 |
| R^2^ | 0.305 | | | 0.311 | | | 0.312 | | |
| ΔR^2^ |  | | | 0.006 | | | 0.001 | | |

| Left nucleus accumbens | | | | | | | | | |
| --- | --- | --- | --- | --- | --- | --- | --- | --- | --- |
| PTSD | -0.001 | -1.07 | 0.287 | -0.001 | -1.07 | 0.287 | -0.001 | -0.78 | 0.438 |
| MetS | - | - | - | 0.001 | 0.01 | 0.991 | -0.001 | 0.26 | 0.800 |
| PTSD x MetS | - | - | - | - | - | - | -0.006 | -0.26 | 0.789 |
| R^2^ | 0.219 | | | 0.219 | | | 0.219 | | |
| ΔR^2^ |  | | | <0.001 | | | <0.001 | | |

PTSD = post-traumatic stress disorder; MetS = metabolic syndrome

A= addition of BMI improved the prediction capabilities of the model beyond PTSD diagnosis in the final model.

*indicates statistical significance at the unadjusted level; **indicates statistical significance following post-hoc correction for multiple testing (p-adjusted threshold= 0.0059) for the final model.

Supplemental Table S6. Effects of the individual metabolic syndrome risk factors on left ventral diencephalon volumes. All models were adjusted for age, sex, scanner site and sequence, alcohol and nicotine use, CTQ and LEC-5 scores.

| DV = Left ventral diencephalon volumes  F (9, 191) = 7.77, R^2^ = 0.27, p <0.001 | | | |
| --- | --- | --- | --- |
| Predictor | Beta-coefficient | T-value | P-value |
| **CAPS-5 total scores** | **-0.003** | **-2.73** | **0.007*** |
| Waist circumference | <0.001 | -0.52 | 0.720 |
| **Fasting glucose** | **0.002** | **-2.09** | **0.036*** |
| Systolic blood pressure | -0.001 | -0.63 | 0.527 |
| Diastolic blood pressure | 0.002 | 0.78 | 0.437 |
| Triglycerides | 0.002 | -0.68 | 0.496 |
| High-density lipoprotein cholesterol | -0.005 | 1.36 | 0.176 |

DV = dependent variable
