# Supplementary material for: Independent effects of posttraumatic stress disorder diagnosis and metabolic syndrome status on prefrontal cortical thickness and subcortical gray matter volumes
Source: Dialogues Clin Neurosci. 2023 Jul 27;25(1):64–74. doi: 10.1080/19585969.2023.2237525 (PMC10375918; doi:10.1080/19585969.2023.2237525)
Supplement: Supplemental Material [file TDCN_A_2237525_SM7131.docx]

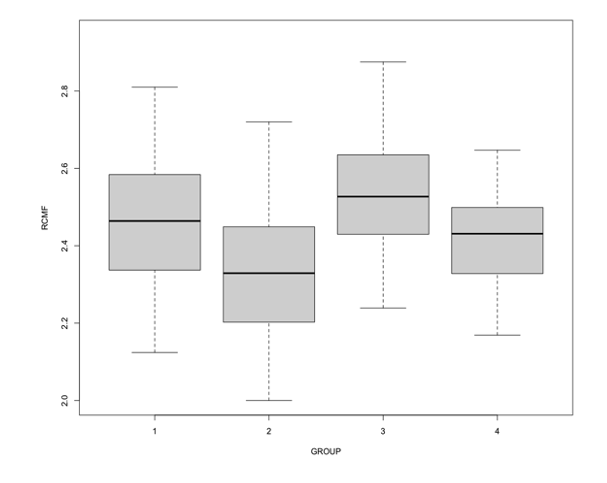


Supplemental Figure S2. Boxplots showing right caudal middle frontal (RCMF) cortical thickness compared between participants stratified according to MetS caseness and PTSD status. Group 1 = patient, - MetS; Group 2 = patient, + MetS; Group 3 = control; - MetS; Group 4 = control; + MetS
