# Supplementary material for: Independent effects of posttraumatic stress disorder diagnosis and metabolic syndrome status on prefrontal cortical thickness and subcortical gray matter volumes
Source: Dialogues Clin Neurosci. 2023 Jul 27;25(1):64–74. doi: 10.1080/19585969.2023.2237525 (PMC10375918; doi:10.1080/19585969.2023.2237525)
Supplement: Supplemental Material [file TDCN_A_2237525_SM7125.docx]

**
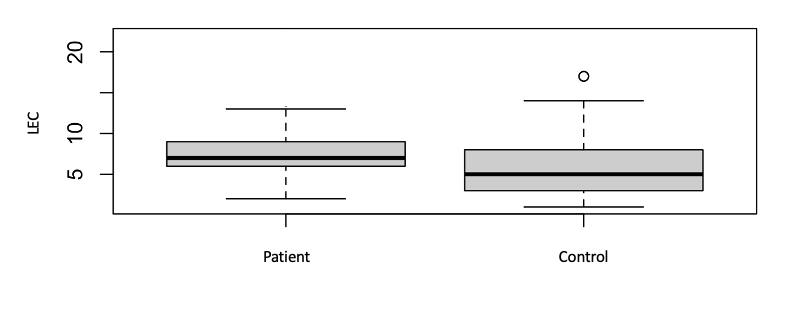
**

**A**

**
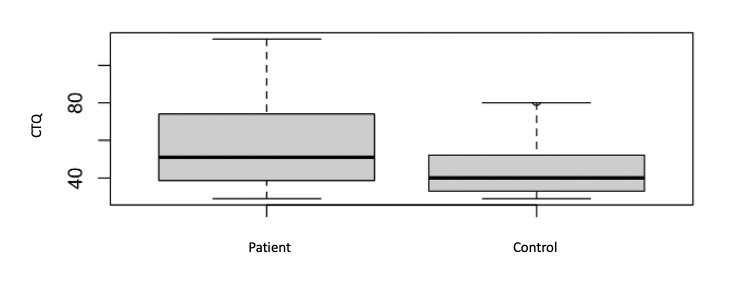
**

**B**

Supplemental Figure S1. LEC-5 number of lifetime trauma exposures (A) and CTQ total scores (B) compared between patients with PTSD and trauma-exposed controls. LEC-5 = Life Events Checklist; CTQ = Childhood Trauma Questionnaire
